# A Novel Antimicrobial Endolysin, LysPA26, against *Pseudomonas aeruginosa*

**DOI:** 10.3389/fmicb.2017.00293

**Published:** 2017-02-27

**Authors:** Mingquan Guo, Chunyan Feng, Jie Ren, Xuran Zhuang, Yan Zhang, Yongzhang Zhu, Ke Dong, Ping He, Xiaokui Guo, Jinhong Qin

**Affiliations:** ^1^Department of Microbiology and Immunology, Institutes of Medical Science, Shanghai Jiao Tong University School of MedicineShanghai, China; ^2^Department of Clinical Medicine, Hangzhou Medical CollegeHangzhou, China

**Keywords:** *Pseudomonas aeruginosa*, bacteriophage, endolysin, LysPA26, antimicrobial agent

## Abstract

The global increase in multidrug resistant (MDR) bacteria has led to phage therapy being refocused upon. A novel endolysin, LysPA26, containing a lysozyme-like domain, was screened against *Pseudomonas aeruginosa* in this study. It had activity against MDR *P. aeruginosa* without pretreatment with an outer-membrane permeabilizer. LysPA26 could kill up to 4 log units *P. aeruginosa* in 30 min. In addition, temperature and pH effect assays revealed that LysPA26 had good stability over a broad range of pH and temperatures. Moreover, LysPA26 could kill other Gram-negative bacteria, such as *Klebsiella pneumonia, Acinetobacter baumannii* and *Escherichia coli*, but not Gram-positive bacteria. Furthermore, LysPA26 could eliminate *P. aeruginosa* in biofilm formation. Our current results show that LysPA26 is a new and promising antimicrobial agent for the combat of Gram-negative pathogens.

## Introduction

Multidrug resistant bacteria, resistant to three or more classes of antimicrobial agent, have been an increasing threat throughout the world in recent years (Li and Vederas, 2009; Young and Gill, 2015). *Pseudomonas aeruginosa*, an opportunistic pathogen, is among the most commonly isolated MDR bacteria in clinical samples. *P. aeruginosa*, with its presence in extensive environmental niches and its adaptive strategy (Lyczak et al., 2000), may be apt to take up plasmids and genes that give multidrug resistance and increase its ability against those antibiotics used clinically (Li et al., 2015; Boss et al., 2016). In addition, the biofilm formation of *P. aeruginosa* is also a form of bacterial resistance mechanism to make it tolerant to conventional antibiotics (del Pozo and Patel, 2007). So, finding a new and effective method to combat the growing number of drug-resistant bacteria is urgent.

The first bacteriophage described in the literature and responsible for the lysin activity was in Twort (1915). Later bacteriophages and bacteriophage-derived products have been employed in the treatment of bacterial infections (Hermoso et al., 2007; Keen, 2012), which has provided an alternative strategy for combating MDR infections. Lysins, phage-lytic or phage-associated enzymes produced by bacteriophages, have natural properties that can cause rapid bacterial cell lysis by efficiently disrupting the peptidoglycan layer (Schuch et al., 2002). Over the past few decades, there has been much research on the lysins controlling bacteria both *in vivo* and *in vitro* (Nelson et al., 2001; Alemayehu et al., 2012). The results showed that lysins could work effectively against Gram-positive bacteria, but were less effective against Gram-negatives due to the protective outer-membrane barrier (Fischetti, 2005). However, if the bacteria were pretreated with OMPs, such as EDTA, Triton X-100, or trichloromethane (CHCl_3_) (Vaara, 1992; Helander and Mattila-Sandholm, 2000), lysins could have an effect on Gram-negative bacteria.

The application of lysins to combat MDR *P. aeruginosa* strains, such as EL188 (Briers et al., 2011), KZ144 (Paradis-Bleau et al., 2007), and OBPgp279 (Walmagh et al., 2012), has been reported. Among these lysins, OBPgp279 was the first reported endolysin that had an antimicrobial activity against *P. aeruginosa* strains in the absence of OMPs (Walmagh et al., 2012). The use of bacteriophages in combating biofilms comprises two strategies, one is to prevent biofilm formation, and the other is to disrupt existing biofilm (Azeredo and Sutherland, 2008). Research by Pires et al. (2011) showed that phage phiIB-PAA2 could destroy *P. aeruginosa* PAO1 biofilm cells, and M4 phage lysate was identified as having the ability to reduce *P*. *aeruginosa* biofilm formation on catheter surfaces (Fu et al., 2010). In this study, bacteriophage JD010 of *P. aeruginosa* was isolated and its endolysin, named LysPA26, was purified. Our results showed that LysPA26 has an intrinsic antimicrobial activity against *P. aeruginosa*. Moreover, it also has an effect on some other Gram-negative bacteria, such as *Escherichia coli* and *Klebsiella pneumoniae*.

## Materials and Methods

### Bacterial Strains and Growth Conditions

The clinical strains *P. aeruginosa, Acinetobacter baumannii, E. coli, K. pneumoniae*, and *Staphylococcus aureus*, isolated from Ruijin Hospital (Shanghai, China), are shown in Supplementary Table S1. All of these isolates were recorded in a computerized database that included source and antimicrobial data. All the bacteria were routinely cultured in LB medium. *E. coli* DH5α and *E. coli* BL21(DE3) were grown in LB broth at 37°C. When needed, 50 ng/ml of kanamycin was added to the growth medium.

### Phage Isolation, Transmission Electron Microscopy, and Genome Analysis

Phage particles were isolated from sewage obtained from Ruijin Hospital (Shanghai, China) by five rounds of plaque purification. Phage JD010 were purified by precipitation with PEG 8000, followed by cesium chloride (CsCl) density-gradient ultracentrifugation. The morphology of phage JD010 was examined after staining with 2% phosphotungstic acid and using a Hitachi 700 transmission electron microscope. The phage DNA was extracted as described elsewhere (Lin et al., 2010) and sequenced with an Illumina MiSeq platform. Using the BLAST at the NCBI^1^, comparative genome analysis of phage JD010 was carried out; the prediction of the conserved protein domain was conducted using BLASTP and the NCBI Conserved Domain Database^2^.

### Overexpression and Purification of LysPA26 Recombinant Protein

*lysPA26* was amplified with the primers lysPA26*-*F/lysPA26*-*R (the *Bam*HI/*Xho*I restriction enzyme sites are shown in bold in the primer sequences in Supplementary Table S2) with JD010 phage genomic DNA as the template. To construct the LysPA26 expression plasmid, the PCR product and vector pET28b were digested with *Bam*HI and *Xho*I restriction enzymes (Fermentas, USA), then the PCR restriction fragment was ligated into the pET28b vector (Supplementary Table S2) at the corresponding restriction sites. After ligation, pET28b-*lysPA26* plasmid was used to transform *E. coli* BL21(DE3), and expression was induced with 1 mM isopropyl-β-D-thiogalactopyranoside for 5 h at 25°C.

### Anti-*P. aeruginosa* Activity Assay

Antimicrobial activity was determined as described previously (Rodriguez et al., 2011) with some modifications. Briefly, *P. aeruginosa* D204 was grown in LB broth at 37°C to exponential phase. Bacterial culture was harvested at 4000 *g* for 5 min and washed once with dilution buffer (20 mM Tris-HCl, pH 8.0). Then, the cell pellet was resuspended in dilution buffer and adjusted OD_600_ to 1.0. For the antimicrobial activity assay, different concentrations of LysPA26 were added into *P*. *aeruginosa* D204 bacterial suspension with or without OMPs (EDTA, Triton X-100, or CHCl_3_). After 30 min of incubation at 37°C, the mixture was serially diluted and plated. To determine the survival rate, residual viable cell numbers (CFUs) on the plate were measured after incubation at 37°C for 24 h. For the negative control, the same volume of buffer was added instead of endolysin LysPA26. The antibacterial activity was expressed as the decrease in viable bacterial counts. Alternatively, the antibacterial activity was presented as the relative inactivation in logarithmic units [= log_10_(N_0_/N_i_), where N_0_ = the number of residual cells (in the negative control) and N_i_ = the number of viable cells counted after incubation with endolysin]. All experiments were performed in triplicate.

### Optimum Conditions for LysPA26 Activity

To study the effect of temperature on the activity of LysPA26, 50 μg LysPA26 was added into 100 μl test bacteria suspension, and the mixture was incubated for 30 min at different temperatures. For the thermal stability assay of LysPA26, the endolysin was incubated at 100°C for 10 min, then the heat-treated LysPA26 was added into the *P. aeruginosa* D204 bacteria suspension with or without 1 mM EDTA. The antibacterial activities were measured as described above. To evaluate the effect of pH on lytic activity, 10 μl LysPA26 (5 mg/ml) was incubated with 90 μl buffers with different pH ranges (50 mM sodium acetate for pH 4.0–6.0 and 20 mM Tris-HCl for pH 7.0–11.0) for 30 min. The effect of saline concentrations on the lytic activity of LysPA26 was tested by adding different NaCl concentrations to cell suspensions for 30 min in 20 mM Tris-HCl buffer, pH 7.0, at 37°C. The residual activity of each treatment group relative to the activity of the control group (100% activity) was determined. All experiments were performed in triplicate.

### Determination of the Lytic Range of LysPA26

To test the lytic zymogram range of LysPA26, *P. aeruginosa, A. baumannii, E. coli, K. pneumoniae*, and *S. aureus* isolates were challenged. All isolates, in mid-exponential phase, were washed and resuspended in dilution buffer (20 mM Tris-HCl, pH 8.0), 50 μg LysPA26 was incubated with the various bacterial species isolates in a final volume of 100 μl, and then serial dilutions of the samples were plated for CFU counting after incubation for 30 min; antibacterial activities were measured as described above. All experiments were performed in triplicate.

### Anti-biofilm Properties

The biofilm eradication assay was carried out by crystal violet staining as described previously (Sass and Bierbaum, 2007). MDR *P. aeruginosa* strain 8328 was cultured in the wells of a polystyrene 96-well plate (BD Falcon) at 37°C for 48 h to allow biofilm formation. The planktonic cells in the culture were discarded and the plate washed with phosphate-buffered saline three times. LysPA26 (100 μg in 200 μl) was added to the plate wells, incubated at 37°C for 2 h, and then washed twice with phosphate-buffered saline. Crystal violet (100 μl 1%) was added and incubated for 30 min; subsequently, 33% acetic acid was added to dissolve the stain, and the optical density was measured at OD_600_. The elimination activity of LysPA26 was also measured by enumerating the reduction of the residual live biofilm cells (Fu et al., 2010). Briefly, after discarding planktonic cells, the biofilm cells were treated with LysPA26 for 2 h. The contents of the treated biofilm-containing wells were mixed fully with a pipetting device, followed by sonication at 42 kHz in a water bath sonicator to make the biofilm cells become planktonic cells. For each experiment, samples were analyzed in triplicate.

### Statistical Analyses

Data were analyzed by a Student’s *t*-test, and a value of *P* < 0.05 was considered statistically significant.

## Results

### Isolation and Characterization of Phage JD010

**FIGURE 1:**
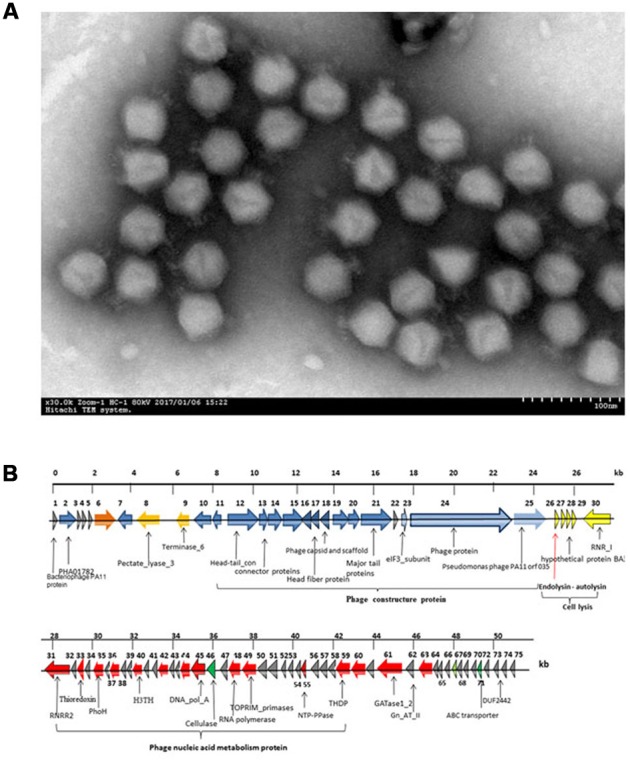
**Characterization of bacteriophage JD010. (A)** Scanning electron microscopy image of phage JD010, negatively stained with 0.2% phosphotungstic acid. The scale bar represents 100 nm. **(B)** Genome organization map of phage JD010. The arrows in the figure indicate the predicted ORF of the phage genome. The red arrow points to the endolysin gene.

A phage, named JD010, was isolated from sewage water in Ruijin Hospital (Shanghai, China). Based on electron microscopy, phage JD010 was classified as a member of family *Podoviridae* with 15 nm tail length and 50 nm head width (**Figure 1A**). Phage JD010 DNA was obtained and sequenced. Phage JD010 has a double-stranded DNA genome of 50609 bp containing 75 ORFs, with a G+C content of 55.5% (**Figure 1B**). BLAST analysis showed that the JD010 genome had sequence similarity with *Pseudomonas* phage PA11, with 89% identity and 83% coverage. ORF 26 of the phage JD010 genome was predicted as the putative endolysin and named LysPA26. BLAST analysis revealed LysPA26 was homologous to the endolysin (ORF033) of *Pseudomonas* phage PA11, with 99% amino acid identity.

### Cloning and Purification of LysPA26

**FIGURE 2:**
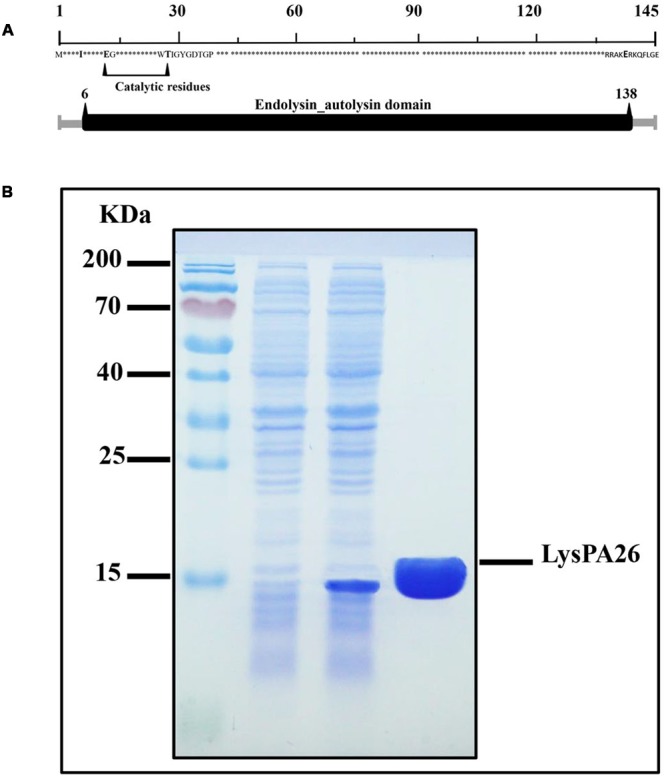
**Expression and characterization of LysPA26. (A)** Schematic diagram of the domain organization of LysPA26. The predicted lysozyme-like domain is marked in dark gray, and involves amino acids 6–138. **(B)** SDS-PAGE gel (15%) of samples from the purification process of LysPA26. Lane 1, molecular size ladder; lane 2, crude lysate extract of BL21(DE3) before induction; lane 3, crude lysate from isopropyl-β-D-thiogalactopyranoside-induced bacteria; lane 4, the purified LysPA26 (21 kDa).

LysPA26 was predicted to belong to the lysozyme-like domain family of superfamily cd00442, which work as peptidoglycan hydrolases. A comprehensive bioinformatics study showed that LysPA26 was composed of a single conserved sequence motif (lysozyme domain), involving the sequence from the 6th to 138th amino acids of the 145 amino acids. The catalytic residues (active sites) of LysPA26 were predicted to be E13 (Glu) and T28 (Thr) (**Figure 2A**). Recombinant LysPA26 was overexpressed and successfully purified from the soluble fraction (**Figure 2B**).

### Antibacterial Activity of LysPA26

**FIGURE 3:**
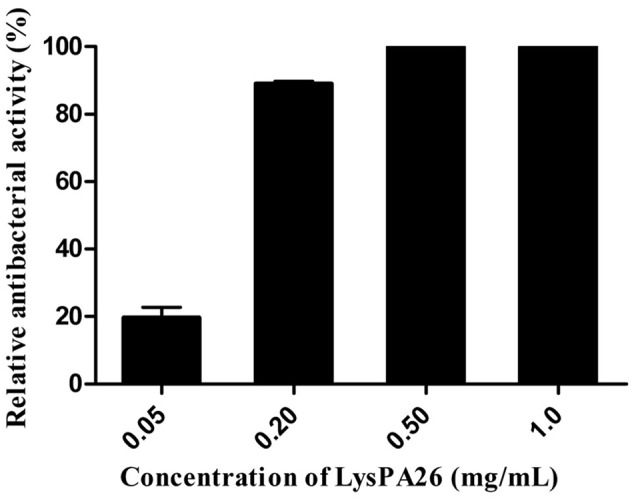
**Antimicrobial activity assay of LysPA26.** Different concentrations of LysPA26 (0.05, 0.2, 0.5, 1 mg/ml) were added to washed *Pseudomonas aeruginosa* D204 cells resuspended in incubation buffer (20 mM Tris-HCl, pH 8.0). The residual cell CFU number of *P. aeruginosa* treated by LysPA26 was counted and presented as the relative inactivation. Error bars represent the standard deviations of three independent assays.

To verify the antibacterial activity of LysPA26, *P. aeruginosa* D204 was challenged with different concentrations of LysPA26. LysPA26 showed efficient bactericidal activity against exponentially growing *P. aeruginosa* D204, and its antibacterial activity was enhanced as the concentration increased (**Figure 3**). It was interesting that LysPA26 could kill *P. aeruginosa* D204 without an OMP. The lytic ability of LysPA26 did not increase any further when the concentration of LysPA26 was more than 0.5 mg/ml. Thus, the endolysin concentration of 0.5 mg/ml was used in the following assays.

**FIGURE 4:**
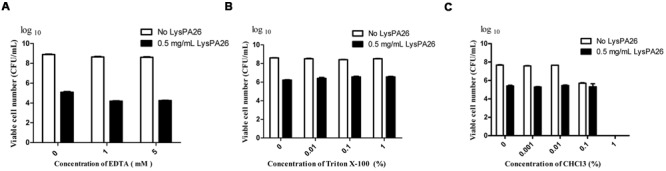
**The synergistic antimicrobial activity assay of LysPA26 and OMPs. (A)** The effect of the concentration of EDTA on the lytic activity of LysPA26. The antimicrobial activity of LysPA26 (0.5 mg/ml) was checked under different concentrations of EDTA (0, 1, 5 mM). **(B)** The effect of the concentration of Triton X-100 on the lytic activity of LysPA26. The antimicrobial activity of LysPA26 (0.5 mg/ml) was checked under different concentrations of Triton X-100 (0, 0.01, 0.1, 1%). **(C)** The effect of the concentration of CHCl_3_ on the lytic activity of LysPA26. The antimicrobial activity of LysPA26 (0.5 mg/ml) was checked under different concentrations of CHCl_3_ (0, 0.001, 0.01, 0.1, 1%). After 30 min of incubation, the residual viable cells were plated and the decrease in viable bacterial numbers measured. Error bars represent the standard deviations of three independent assays.

With pre-treatment by OMPs, such as EDTA, Triton X-100, or CHCl_3_, lysin can effectively destroy Gram-negative bacteria as reported. In order to test the effect of OMPs on the bactericidal activity of LysPA26, EDTA, Triton X-100, and CHCl_3_ were selected to individually pretreat *P. aeruginosa* D204, and antibacterial activity was expressed as the decrease in viable bacterial counts (**Figures 4A–C**). Different concentrations of OMPs were tested to determine whether they had synergistic effects on the antimicrobial activity of LysPA26. The results showed the EDTA could enhance the lytic ability of LysPA26 against *P. aeruginosa* D204 in a certain range, but Triton X-100 and CHCl_3_ could not. Thus, 1 mM EDTA was selected for use as the OMP in the following experiments, where needed.

### Determination of Optimal Conditions for the Activity of Endolysin LysPA26

**FIGURE 5:**
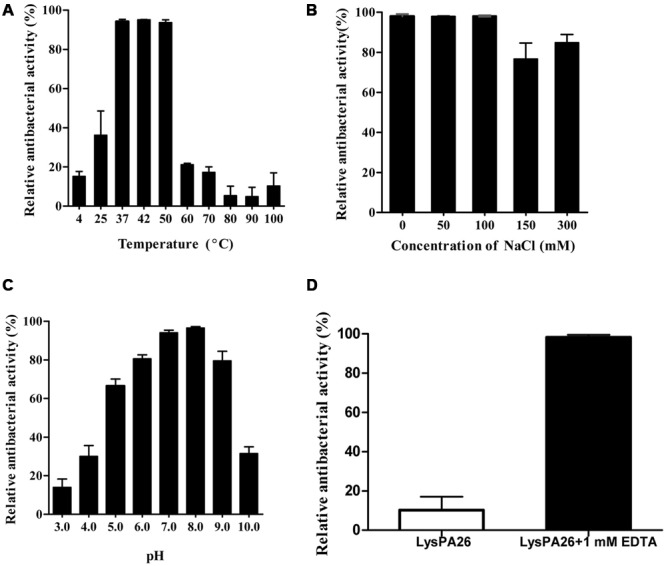
**Influence of temperature, NaCl and pH conditions on the bactericidal activity of LysPA26. (A)** The effects of temperature on the lytic activity of LysPA26. LysPA26 (0.5 mg/ml) was added to test bacterial suspensions and incubated for 30 min at different temperatures to determine the bactericidal assay. **(B)** The influence of saline concentration on the bactericidal activity of LysPA26. LysPA26 (0.5 mg/ml) was added to test bacterial suspensions under different NaCl concentrations to determine the antimicrobial activity. **(C)** The influence of pH on the lytic activity of LysPA26. LysPA26 (0.5 mg/ml) was incubated with buffers with different pH values (50 mM sodium acetate for pH 4.0–6.0 and 20 mM Tris-HCl for pH 7.0–11.0) for 30 min to determine the antimicrobial activity. **(D)** Thermo-stability of LysPA26. LysPA26 (0.5 mg/ml) was incubated at 100°C for 10 min before determining the antimicrobial activity in the absence of EDTA or with 1 mM EDTA. Bactericidal activity is presented as the relative inactivation. Error bars represented the standard deviations of three independent assays.

The optimal conditions for the bactericidal activity of LysPA26, such as temperature, pH, and saline concentration, were tested. Temperature was the key factor affecting the bactericidal activity of endolysin. As shown in **Figure 5A**, LysPA26 exhibited high antibacterial activity from 37 to 50°C. The antibacterial activity of LysPA26 decreased when the temperature was lower than 25°C or higher than 60°C. As for the effect of saline concentration on the activity of LysPA26, NaCl was selected for the test. The results showed that a NaCl concentration below 150 mM did not show a significant effect on the activity of LysPA26. However, when the concentration was high enough, up to 300 mM, it could reduce the efficacy of LysPA26 (**Figure 5B**). As for the effect of pH on activity of LysPA26, it was efficiently active from pH 6.0 to 8.0, and the optimal pH was 8.0 (**Figure 5C**). The results showed the LysPA26 has a good pH stability in the antibacterial activity assay.

We also checked the thermostability of LysPA26, it was heat-treated at 100°C for 10 min before being used to challenge exponentially growing *P. aeruginosa* D204; the cells were treated with EDTA or were untreated. For the challenged bacteria without EDTA treatment, the activity of LysPA26 decreased to less than 20%. However, when treated with the EDTA, LysPA26 still maintained over 90% of its activity (**Figure 5D**). This may be attributed to the stability of LysPA26 catalytic sites and the reliable permeabilization of EDTA, which makes the peptidoglycan fully exposed.

### LysPA26 Shows an Antibacterial Spectrum against more MDR Gram-Negative Species Besides its Host Strain

**FIGURE 6:**
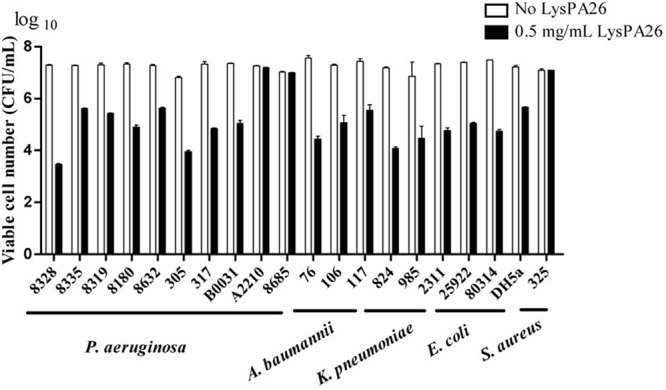
**The antimicrobial spectrum of the LysPA26.** Bacteria, including both Gram-negative and Gram-positive bacteria, were examined. For all the challenged isolates, 0.5 mg/ml LysPA26 was added into the bacteria suspension after 30 min incubation at 37°C. The antibacterial activity was expressed as the decrease in viable bacterial numbers. Error bars represent the standard deviations of three independent assays.

To further estimate the zymogram range of LysPA26 against clinical MDR strains, clinical samples including Gram-negative (*A. baumannii, K. pneumoniae, P. aeruginosa*, and *E. coli*) and Gram-positive (*S. aureus*) bacteria were tested. LysPA26 exhibited high antibacterial activity against the tested Gram-negative bacteria (**Figure 6**). It was apparent that the antibacterial spectrum of LysPA26 was broader than phage JD010. Of note, LysPA26 could not kill *P. aeruginosa* A2210, which is the host of JD010. A methicillin-resistant *S. aureus* isolate was tested to detect the antibacterial activity of LysPA26 against Gram-positive bacteria. The results showed no antibacterial activity against the strain.

### LysPA26 Is able to kill *P. aeruginosa* in Biofilms

**FIGURE 7:**
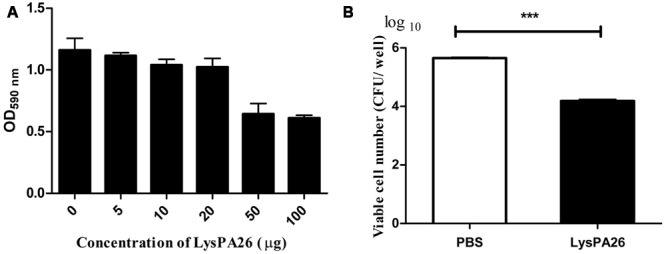
**Biofilm eradication ability of LysPA26.** Biofilm was grown on a polystyrene plate. **(A)** Biofilm eradication ability was determined by optical density analysis. **(B)** Eradication ability of LysPA26 was expressed as the discrepancy of the number of viable cells in the different treatments. ^∗∗∗^ Indicates significant difference (*P* < 0.001). Error bars represent the standard deviations of three independent assays.

*Pseudomonas aeruginosa* is one of the most common biofilm-forming bacteria. We designed experiments to test whether LysPA26 had the ability to destroy *P. aeruginosa* biofilm. *P. aeruginosa* 8328 was attached to a plate for 48 h to form biofilm before adding LysPA26. We found that the LysPA26 had the ability to efficiently destroy existing biofilm cells. The elimination ability of biofilm was dose dependent; the optical density (OD_600_) staining of the biofilms was reduced significantly upon addition of LysPA26 up to 50 μg (**Figure 7A**). Approximately 1–2 log in the number of viable counts of biofilm cells in the plate wells was disrupted by LysPA26 (100 μg) (**Figure 7B**). Combined with the other results, the elimination efficiency for the biofilm cells was comparatively lower than that for the planktonic cells.

## Discussion

Multidrug resistant bacteria, such as *P. aeruginosa, A. baumannii, K. pneumonia* and *S. aureus*, have been an increasing threat throughout the world in recent years. The emergence and dissemination of MDR strains of various bacteria species has posed a big challenge for effective clinical treatment. This constantly emerging phenomenon of MDR bacteria has renewed interest in the use of phages as antimicrobial agents (Pires et al., 2016).

There has been much research of phage lysins, and most of it has been with Gram-positive pathogens. Phage lysins have been shown to be ineffective against Gram-negative bacteria by direct exogenous application, because of the protective outer-membrane barrier in these bacteria (Fischetti, 2005, 2010). If cells were pretreated with an OMP, such as EDTA or Triton X-100, the lysins can destroy Gram-negative bacteria (Briers et al., 2014). Nevertheless, recent published data supports the idea that some natural lysins have intrinsic antibacterial activity against Gram-negative bacteria in the absence of an OMP, for example endolysin LysAB21 and PlyF307 from *A. baumannii* phage (Lai et al., 2011; Lood et al., 2015), and endolysin OBPgp279 from *Pseudomonas fluorescens* phage OBP (Walmagh et al., 2012).

Basic Local Alignment Search Tool analysis of publically available data confirmed that LysPA26 has only one homolog, gene ORF033 of *Pseudomonas* phage PA11 (Kwan et al., 2006), but its features have not been reported. Unlike some other Gram-negative endolysins possessing an N-terminal binding domain and a C-terminal catalytic domain (Briers et al., 2011; Walmagh et al., 2013), LysPA26 is a single domain endolysin. In this study, we found that 0.5 mg/ml LysPA26 could kill about 4 log unit bacteria in 30 min when incubated with 10^8^ exponential cells of host bacteria *P. aeruginosa* D204, and the reaction was in the absence of EDTA. When the challenged cells were pretreated by the addition of 1 mM EDTA, the bacteria were more sensitive. For the same reaction, more than 1 log cells were killed by endolysin LysPA26, suggesting that EDTA enhanced the antibacterial capacity of LysPA26, which has been reported previously (Lim et al., 2014; Oliveira et al., 2014). However, when 5 mM EDTA was added, the greater addition of EDTA did not enhance the LysPA26 to further reduce the number of viable *P. aeruginosa* D204 cells, a similar effect was described with SPN9CC endolysin from SPN9CC phage against *Salmonella Typhimurium*, which could kill intact Gram-negative bacteria in the absence of EDTA (Lim et al., 2014).

The pH test for endolysin LysPA26 showed that LysPA26 was stable at a broad pH range (5.0–9.0), which is a good feature required for promising antimicrobial agents. Its optimal pH was around 8.0. Temperature tests of LysPA26 showed that LysPA26 retained high activity (relative antibacterial activity > 90%) against *P. aeruginosa* D204 at 50°C. Unexpectedly, we found that LysPA26 still exerted a high bactericidal activity against the outer-membrane permeabilized isolates under heat treatment at 100°C. Some Gram-negative bacterial endolysins in previous studies showed good thermostability, for example endolysins KZ144 and EL188 from *Pseudomonas* phage could retain high activity at 50°C (Briers et al., 2007). PVP-SE1gp146, described as the first thermo-resistant Gram-negative phage endolysin from *P*. *aeruginosa* PAO1, could maintain activity at temperatures up to 90°C (Walmagh et al., 2012). BLAST analysis revealed that there was no significant similarity between PVP-SE1gp146 and LysPA26, although both of them have thermo-resistance. We presumed that for both of them, their thermo-resistance can likely be attributed to their stable conformational structure.

In our results, LysPA26 could not only kill *P. aeruginosa* efficiently, but also combat other Gram-negative species, such as *E. coli*, and *K. pneumoniae*, which are among the most drug-resistant Gram-negative pathogens. However, it was ineffective against Gram-positive bacteria, such as *S. aureus.* We suspect that the peptidoglycan layer of Gram-positive strains is not susceptible to endolysin LysPA26. This characteristic makes LysPA26 a promising candidate for selectively treating Gram-negative species.

Bacterial biofilm is a type of survival strategy for resisting the host defense mechanism or sub-optimal environmental conditions (Donlan and Costerton, 2002). Due to the increased tolerance to antibiotic treatment, many clinical complications associated with numerous biofilm-forming pathogens are very difficult to treat (Costerton et al., 1999). Bacteriophages and bacteriophage-enzyme-based strategies for prevention and eradication of biofilm bacteria have been extensively studied and proposed to be a promising bio-control method against bacteria (Xavier et al., 2005). The biofilm-disrupting activity experiment in this study showed that LysPA26 exhibited the ability to disrupt *P. aeruginosa* in biofilm form and degraded biofilms in a concentration-dependent manner.

Although further investigation and additional experiments are needed to explain the efficacy and the lytic mechanism of LysPA26, it is undoubtable that LysPA26 is a novel and promising agent for directly combating MDR *P. aeruginosa* in the planktonic form, as well as in the biofilm form. This endolysin could be a desirable candidate for use as a bio-control tool against Gram-negative pathogen infection.

## Author Contributions

JQ, XG, and MG conceived and designed the study. CF, JR, XZ, YaZ, and KD carried out the experiments. YoZ and PH analyzed the data. All authors contributed to the writing of the manuscript.

## Conflict of Interest Statement

The authors declare that the research was conducted in the absence of any commercial or financial relationships that could be construed as a potential conflict of interest.

## Footnotes

1 http://www.ncbi.nlm.nih.gov/BLAST/

2 http://www.ncbi.nlm.nih.gov/Structure/cdd/wrpsb.cgi

## Abbreviations

BLAST: Basic Local Alignment Search Tool
CFU: colony forming unit
EDTA: ethylene diamine tetraacetic acid
MDR: multidrug resistant
NCBI: National Center for Biotechnology Information
OMP: outer-membrane permeabilizer
ORF: open reading frame
