# Supplementary material for: A Novel Antimicrobial Endolysin, LysPA26, against Pseudomonas aeruginosa
Source: Front Microbiol. 2017 Feb 27;8:293. doi: 10.3389/fmicb.2017.00293 (PMC5326749; doi:10.3389/fmicb.2017.00293)
Supplement: Supplementary file 1 [file Table_1.PDF]

Table S1 MDR Bacteria strains used in this study

Strains	Relevant characteristic
*P. a*eruginosa	
D204	NMDR strain
305	NMDR strain
317	NMDR strain
8328	MDR strain
8335	MDR strain
8685	MDR strain
8180	MDR strain
8319	MDR strain
A2210	MDR strain
8632	MDR strain
B0031	MDR strain
A. baumannii	
76	MDR strain
106	MDR strain
117	NMDR strain
K. pneumoniae	
824	MDR strain
985	NMDR strain
2311	MDR strain
E. coli	
25922	NMDR strain
80314	MDR strain
DH 5α	-
S. aureus	
325	MDR strain
