# Supplementary material for: A Novel Antimicrobial Endolysin, LysPA26, against Pseudomonas aeruginosa
Source: Front Microbiol. 2017 Feb 27;8:293. doi: 10.3389/fmicb.2017.00293 (PMC5326749; doi:10.3389/fmicb.2017.00293)
Supplement: Supplementary file 2 [file Table_2.PDF]

Table S2 primers used in this study

primer	Sequence (5’-3’)
D204-F	CGGGATCCGCGAACGAATAATATCGACGC
D205-R	CCGCTCGAGTTCTCCAAGGAATTGTTTAC
LysAB-209-F	CGGGATCCGATTCTGACTAAAGACGGGTT
LysAB-209-R	CCGCTCGAGTAAGCTCCGTAGAGCGCGTT
